# Post-Consumer Recycled PET: A Comprehensive Review of Food and Beverage Packaging Safety in Brazil

**DOI:** 10.3390/polym17050594

**Published:** 2025-02-24

**Authors:** Carolina Soares Marcelino, Vitor Emanuel de Souza Gomes, Luís Marangoni Júnior

**Affiliations:** Department of Food Engineering and Technology, School of Food Engineering (FEA), State University of Campinas (UNICAMP), Campinas 13083-862, Brazil

**Keywords:** polymeric packaging, PET bottles, circular economy, contaminants, non-intentionally added substances

## Abstract

Polyethylene terephthalate (PET) is widely used in the food and beverage packaging sector due to its chemical and mechanical properties. Although PET is a fossil-based polymer, its recyclability significantly contributes to reducing the environmental impacts caused by excessive plastic consumption. However, the growing demand for post-consumer recycled PET (PET-PCR) food packaging has raised concerns about the efficiency of decontamination processes involved in recycling this material. This review initially addresses PET synthesis processes, highlighting injection stretch blow molding as the predominant technique for packaging production. It then discusses reverse logistics as a strategy to promote sustainability through the recovery of post-consumer packaging, such as plastic bottles. This review examines mechanical and chemical recycling methods used in PET-PCR production, food safety requirements including positive lists of permitted substances, contaminant migration limits, non-intentionally added substances (NIASs), and updated criteria for the National Health Surveillance Agency (ANVISA) of food-grade PET-PCR resins. Finally, the review explores future prospects for using PET-PCR in the food and beverage packaging sector, assessing its environmental impacts and potential technological advancements to enhance its sustainability and safety.

## 1. Introduction

The growing concern for the preservation of natural resources and the pursuit of sustainability have driven demand in the packaging industry for easily recyclable materials, particularly those derived from fossil sources [1,2,3]. Among these materials, polyethylene terephthalate (PET) stands out for its excellent physicochemical properties, making it widely used in food packaging production.

PET combines mechanical and chemical strength with optical versatility, appearing either transparent (amorphous) or translucent (semi-crystalline) [4]. This characteristic is particularly valued as it allows consumers to see the contents of the packaging [5]. Additionally, PET is lightweight compared to glass, impact-resistant, and retains its elasticity during processing [6]. Its chemical inertness surpasses that of other plastics, making it ideal for contact with food and beverages [7]. It is commonly used in packaging for sauces, juices, oils, and bottles for soft drinks and water [8].

The production of post-consumer recycled PET (PET-PCR) bottles for food contact starts with PCR resin, sourced from used and discarded bottles that are reintroduced into the production chain through validated recycling and decontamination processes [9,10].

Research on the recycling rate in Brazil is conducted by the Brazilian Association of the PET Industry (ABIPET) and considers three distinct industrial groups: recyclers, who exclusively recycle PET material (26%); integrators, who both recycle and utilize recycled PET (13%); and applicators, who solely use recycled PET (61%). In Brazil, the recycling rate of PET packaging reached 56.4% in 2021 (calculation based on collection weights), marking a 15.4% increase compared to 2019. This growth in recycling outpaced the rise in the consumption of virgin resin, which was 12.4% during the same period. This outcome reflects the strong establishment of a circular economy in the sector, which aims to add value to recycled PET in various industrial products. The 12th PET Recycling Census in Brazil revealed that the pre-form and bottle industry, mainly for soft drinks and water, leads the consumption of PET-PCR, accounting for 29%. The textile sector follows with 25%, while the packaging industry for solids represents 17% of the total consumed [11].

However, despite the growing demand in the food and beverage sector, there are concerns regarding the efficiency of PET decontamination during recycling. Contaminants present in the packaging can remain in the recycled materials, leading to the migration of compounds into food and beverages. Therefore, it is essential to establish a methodology that ensures the adequacy of the recycling process and the safety of the packaged products [10,12].

Recently, Bezeraj et al. published a comprehensive review that addresses the challenges faced by the mechanical PET recycling industry and emphasizes the importance of quantifying the multi-scale characteristics of the synthesis and recycling of this material [13]. Additionally, other reviews and research articles on the safety of recycled PET for food contact have also been published, with a broader focus [14,15,16,17,18,19]. However, our review aims to specifically focus on the Brazilian market for recycled PET packaging intended for food and beverage contact. Thus, the objective of this work is to present a review of the PET recycling chain for food and beverage contact, highlighting potential contaminants and the necessary methodologies to validate the recycling process according to regulatory standards.

## 2. Production of Virgin PET

**Figure 1:**
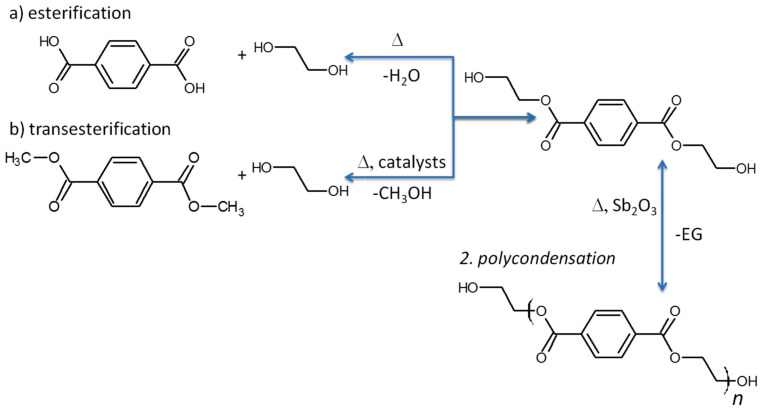
Synthesis reactions of PET: (**a**) esterification reaction and (**b**) transesterification reaction [5]. Copyright 2025, with permission from Elsevier.

The production of virgin PET can be carried out through two main processes. In the first method, illustrated in Figure 1a, direct esterification occurs between terephthalic acid (TPA) and ethylene glycol (EG). During this reaction, which takes place under pressures of 2.7 to 5.5 bar and elevated temperatures (220 to 260 °C), a continuous distillation process is used to remove the generated water. TPA itself acts as a catalyst in the esterification reaction, and the high temperatures facilitate its solubilization in EG. However, as TPA is consumed, it is necessary to add metal catalysts to continue the reaction [20].

The second method begins with the transesterification reaction (Figure 1b) between dimethyl terephthalate (DMT) and an excess of ethylene glycol (EG), at lower temperatures (150 to 200 °C) and in the presence of catalysts such as magnesium and zinc. This process produces bis(2-hydroxyethyl)-terephthalate (BHET) as the initial product. After this step, the excess EG and methanol are removed through continuous distillation. Next, the BHET undergoes polycondensation at a temperature of 270 to 280 °C (close to the melting point of PET) and a pressure of 10 to 50 Pa, resulting in the formation of the PET polymer [20]. Catalysts such as Sb_2_O_3_, Sb_2_O_5_, and GeO_2_ are commonly used during this phase of the process [21,22]. After polymerization, the material is cooled to stabilize the polymeric structure and then fragmented into small particles known as pellets, which serve as raw material for molding processes [23].

Since PET is a hygroscopic material, it is crucial to dry it before extrusion to avoid residual moisture that can cause hydrolysis reactions. Drying should occur at temperatures between 120 °C and 180 °C for a period of 6 to 24 h, resulting in a moisture content of 0.005% by weight. However, the temperature should not exceed 180 °C to prevent thermal degradation of the PET. Finally, extrusion is carried out using one or two screw systems, assisted by barrel heaters and shear heating, to melt the polymer pellets. The melted material is then directed into a mold to achieve the desired shape [5].

## 3. Injection and Blow Molding

Injection and blow molding for the production of rigid PET packaging begins with the injection of the material, resulting in small tubular plastic structures known as preforms. This injection molding process typically occurs at temperatures between 250 °C and 280 °C, with the mold cooled to 8 °C and a cycle time of 30 s [24]. After injection molding, the preforms are heated to temperatures between 100 °C and 110 °C, making them more pliable and ready for the next stage. At this point, the preforms are transferred to the stretch blow molding machine, where air is introduced to shape them into the desired final form of the packaging [25].

**Figure 2:**
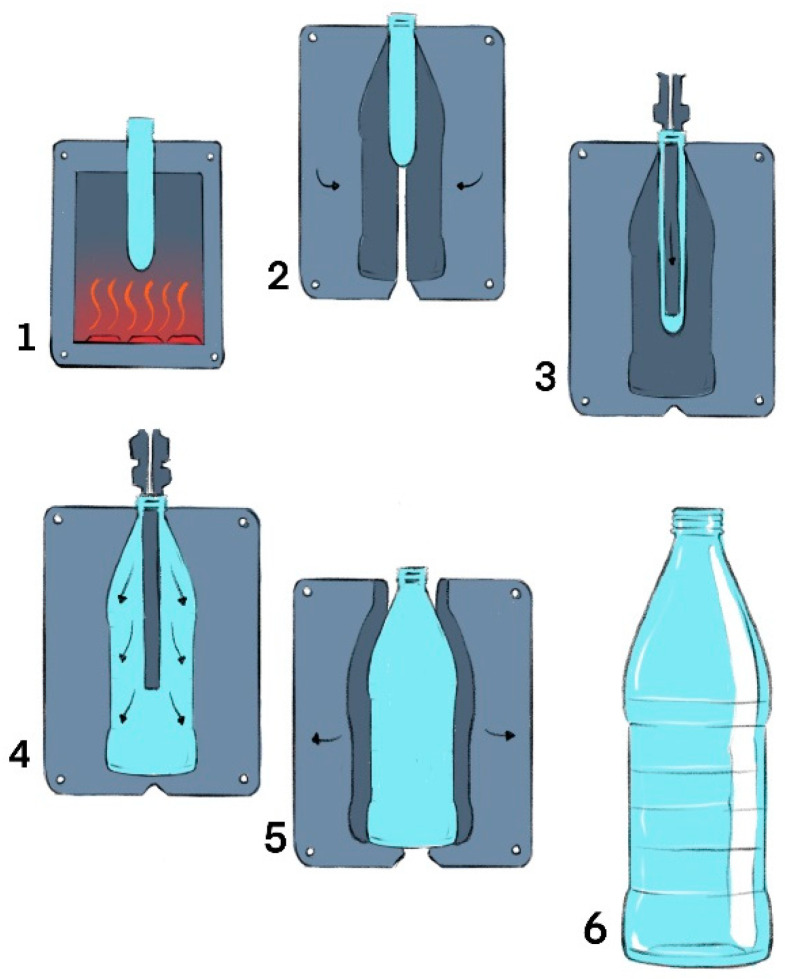
Injection and blow molding processes of PET bottle. The polymer is melted (**1**), the molten polymer is fed through nozzles into heated cavities with core pins, forming pre-forms by injection molding (**2**), the pre-forms are shaped in a single injection cycle (**3**), compressed air is introduced into the pre-form, inflating it to acquire the final shape of the bottle (**4**), the blow mold opens, and the core rod rotates to the ejection position (**5**), and the bottle is removed from the core rod (**6**).

In summary, as illustrated in Figure 2, the blow molding process occurs when the preforms are inserted into the mold of a bottle or flask. During this stage, the pieces undergo two types of deformation: radial, due to the internal air pressure, and axial, from the action of a stretching rod. Once the bottles are formed, they can be filled with a variety of products, commonly used for carbonated beverages and mineral water [26]. Blow molding takes place at an optimal temperature of around 105 °C, allowing the PET to achieve the desired orientation [27]. Additionally, the shape of the bottles is determined by the pressure provided by high-pressure compressors [28].

## 4. Reverse Logistics

After the use and disposal of packaging made from virgin material by the final consumer, the reverse logistics process begins [29]. At this stage, consumers take on the role of suppliers, and their participation in post-consumer packaging recovery programs is crucial for the effectiveness of reverse logistics. They must carry out proper collection and sorting to ensure recycling [30]. Reverse logistics is essential for the circular economy as it enables the recovery, reuse, and recycling of resources, reducing waste, demand for raw materials, and environmental impacts. This process closes the loop in supply chains, extending the lifespan of resources and promoting sustainability [31].

**Figure 3:**
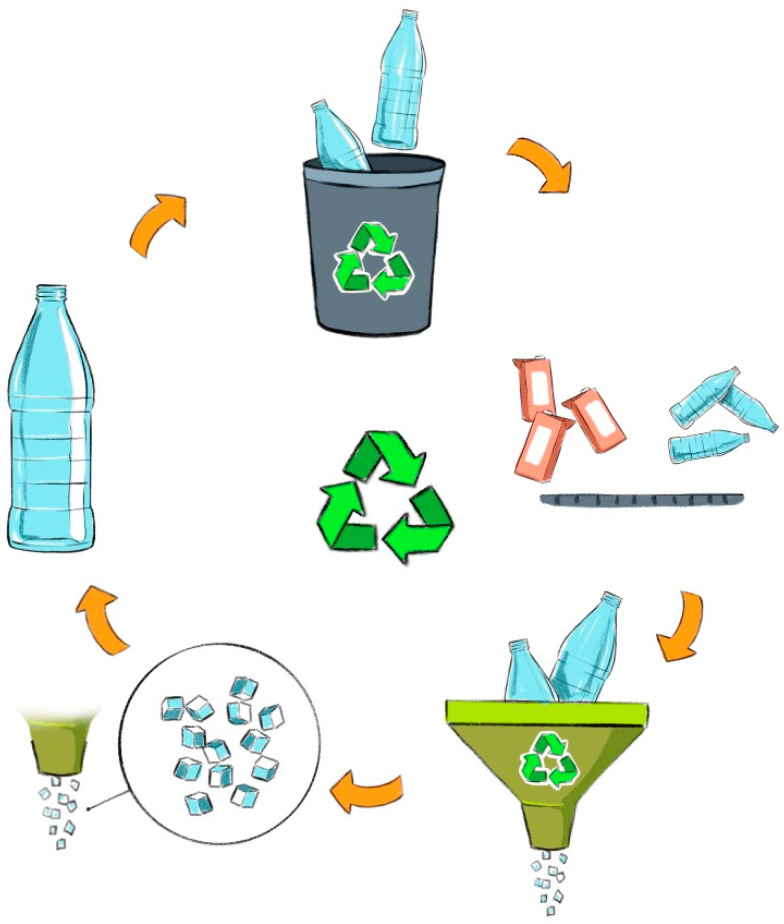
Reverse logistics scheme for PET recycling.

As illustrated in Figure 3, reverse logistics management involves several stages: generation and management of waste at the source, collection, transportation, processing, transformation, and final disposal. Post-consumer PET packaging collected and sorted at the source represent the first link in the reverse logistics chain. Information about this waste is essential to determine the type of material available for recycling, defining the volume to be recycled and mitigating contaminants that need to be removed during the recycling process to ensure safe use as materials in contact with food and beverages [32].

The collection of PET bottles in Brazil is predominantly carried out through a system that includes both the formal and informal sectors, with waste pickers playing a key role [33]. These workers, who often operate independently, are responsible for recovering a significant portion of discarded PET bottles. They traverse streets, landfills, and collection points, contributing not only to environmental preservation but also providing a source of income for many families. However, this dynamic can impact the quality of the collected bottles, as mixing them with other waste during collection may introduce unwanted contaminants [33,34,35,36,37].

Additionally, an analysis of bottle designs in Brazil may reveal non-recyclable components in some packaging. These elements can complicate the recycling process, requiring further separation to ensure that only recyclable material reaches recycling facilities. PET bottles often need to be sorted by type and color before recycling, which is a critical step to maintain the quality of the final material. In terms of logistics, Brazil faces significant challenges, primarily due to vast transportation distances, which not only increase operational costs but also generate additional environmental burdens because of the carbon emissions associated with long-distance transport [34,37].

Lastly, the management of the PET recycling chain in Brazil could benefit from increased regulation and public incentives to improve selective collection and promote awareness of proper disposal methods. Including the informal sector in waste management policies, along with initiatives that prioritize environmental education, can enhance the efficiency of the collection system and, consequently, the quality of recycled PET [34,36,37]. These issues are discussed in various academic publications and reports that could enrich a more in-depth analysis of PET recycling in the Brazilian context.

## 5. Mechanical and Chemical Recycling of PET

Recycling of PET can be carried out in four ways: primary, secondary, tertiary, and quaternary. Primary recycling, the simplest process, involves the recovery of waste from the industry, known as pre-consumer waste or production scrap, which has a high level of purity. It may also include post-consumer waste that has undergone rigorous selection [38,39]. The recovered plastics retain performance characteristics similar to virgin plastics and are typically reprocessed mechanically, usually through high-temperature extrusion under pressure to restructure the polymer chains [26].

Mechanical recycling is the most widely used technique for PET due to its technical feasibility, especially in relation to the relatively low cost and high yields compared to chemical recycling methods which are more expensive. The steps in this process include collecting post-consumer material, followed by sorting to separate PET from other materials present [40,41]. This separation can be performed manually or automatically, using methods such as induction sorting, X-rays, and infrared sensors, often combined to maximize purity, which can reach up to 95% [29,42]. After sorting, the material is shredded into small pieces known as flakes to facilitate transportation and improve density [42]. Next, the material undergoes milling, refining it into even finer particles, which increases the surface area and ensures uniformity. This process requires equipment such as mills or grinders [29].

**Figure 4:**
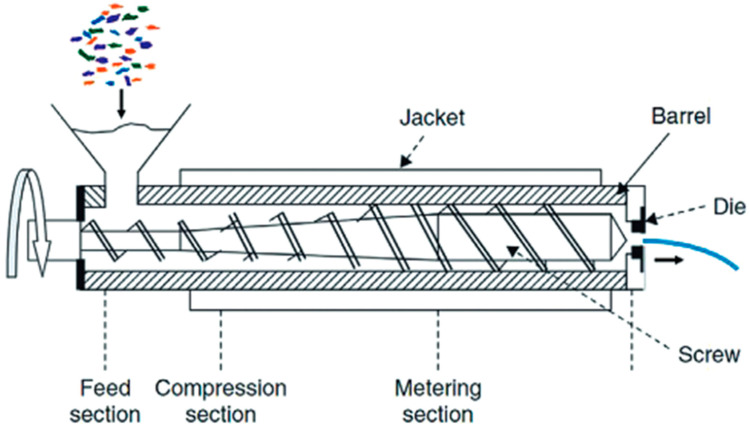
Illustration of the mechanical recycling extrusion process [44]. Copyright 2025, with permission from John Wiley and Sons.

The flakes are washed in water at 85 °C using a solution containing 1% by weight of sodium hydroxide (NaOH) and detergent for 15 min [43]. After washing, the flakes are dried until they reach less than 0.1% moisture content, making them ready for reprocessing [42]. The extrusion process, illustrated in Figure 4, is the most commonly used method in industrial mechanical recycling. It operates through heat and rotating screws that facilitate the plasticization of the material. However, increased temperature and shear can cause degradation of the polymer chains, reducing their length and altering mechanical properties. The primary effect of this degradation is the formation of radicals along the polymer chain, induced by peroxide radicals generated by oxygen, leading to chain breakage, decreased viscosity and yellowing [44].

Additionally, the intensity of polymer degradation can be controlled by the temperature and the screw rotation speed, which directly impacts the quality of the final product. Another important point is that the PET chains can intertwine, which affects the quality of the recycled material. In large-scale recycling operations, the increase in viscosity caused by this intertwining can become problematic, as high torques may damage the processing equipment [44]. However, the reprocessing of polymers has been optimized with advancements in extrusion technologies, which incorporate sections for degassing and filtration of the extrudate, enhancing the quality of the polymer melt [45,46]. Degassing allows for the release of volatile compounds, reducing degradation and improving odor, thereby increasing the value of the recycled material. Simultaneously, filtration eliminates larger contaminants, such as dust and gel, promoting greater homogeneity and enhancing the mechanical and optical properties [44,46]. The filters used are selected based on the type of contamination and may include options such as sliding plates, woven screens, or filter cartridges [47]. The main advantages of the extrusion recycling process include cost reduction, large-scale production capacity, the elimination of solvents, and versatility in processing different polymers [44].

Although PET recycling in Brazil is primarily mechanical, there is the possibility of chemical recycling; however, this approach is still largely unexplored by the industry. Currently, scientific research is focused on this area, aiming to deepen the understanding and applications of chemical recycling to enhance the reuse of this material.

**Figure 5:**
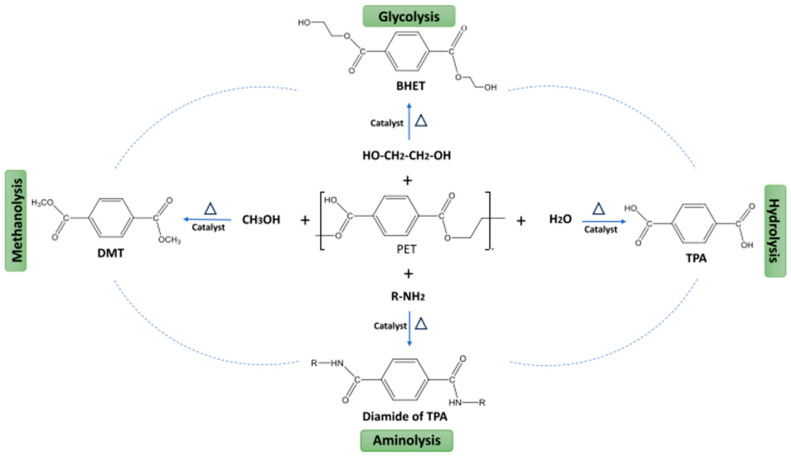
Routes of chemical recycling of PET [49]. Copyright 2025, with permission from Elsevier.

The chemical recycling of PET, illustrated in Figure 5, involves reactions that decompose its molecular structure into high-quality monomers, enabling the creation of new products. The process begins with depolymerization, where heat and excess methanol are applied in the presence of a catalyst, often zinc acetate or magnesium acetate [48]. This stage results in the breakdown of polymer chains into monomers. Glycolysis is one of the main depolymerization pathways aimed at obtaining BHET and EG, utilizing organocatalysts, metal chlorides, and acetates, the choice of which impacts the efficiency and cost of the process [29].

Next, in the purification stage, the contaminants are removed. Finally, a new polymerization is conducted, which again applies high temperatures and a catalyst to rebuild the polymer chains of the recycled material [29]. Glycolysis, which utilizes EG to produce BHET through transesterification, is the most commonly used methodology in the industry due to its viability. The most common catalysts for this reaction include antimony (III) oxide and antimony acetate [50].

If the materials are not properly prepared for recycling, whether due to high levels of contamination or a mix of plastics, energy or quaternary recycling through pyrolysis may be a viable alternative [44].

After the production of the PET-PCR resin, it is essential to measure the intrinsic viscosity (IV), which reflects the molecular weight and is directly associated with the mechanical properties and applications of PET-PCR [51]. A study conducted in the United States on PET films with various proportions of PET-PCR revealed that a higher percentage of recycled resin results in lower IV. This can be explained by the presence of contaminants that generate acidic components, catalyzing the hydrolytic cleavage of the ester bond and forming terminal carboxylic acid and hydroxyl ester groups, which shorten the polymer chains and decrease the IV [52]. Additionally, a study evaluating bottles made from PET-PCR compared to virgin PET indicated that the recycled bottles are more susceptible to thermal and hydrolytic degradation, possibly due to the presence of contaminants that generate acids, acting as catalysts for the cleavage of the ester bond in PET. This contamination, combined with retained moisture, results in chain breakage and, consequently, a reduction in IV and molecular weight of the PET-PCR resins [24].

It is worth noting that a reduction in IV may be acceptable for textile applications, but it should be avoided in rigid packaging, such as bottles and jars. To maintain the IV during extrusion, a solid-state polycondensation step can be applied before the melt molding process [53]. In packaging applications, the often-desired IV ranges between 0.7 and 1.04 dL/g [25]. Finally, a study emphasized the importance of a high IV, greater than 0.8 dL/g, to ensure that the length of the polymer chains is adequate for bottle formation [54]. It is important to note that, due to the specific IV range required for bottles, the recycled PET used in this category in Brazil accounts for 29% of the total. Additionally, 24% of recycled PET is allocated to the textile sector, 17% is used for the production of thermoformed products, 11% for strapping tapes, and 19% for other chemical products [11]. This distribution highlights the versatility of recycled PET and its application across various industries.

Improvements in PET recycling are the subject of extensive studies, particularly regarding the number of extrusions that PET can withstand before becoming unsuitable for (re) manufacturing [44]. After three recycling cycles, changes in the properties of the material are minimal due to the slow degradation that occurs, which is influenced by the size of the polymer chains [55]. To enhance the mechanical properties and add value to recycled PET, additives are often used [44,56]. For example, tin mercaptide and lead phthalate are effective in mitigating thermal oxidation. Additionally, stabilizers and radical scavengers, such as organic phosphates, play a crucial role in protecting the polymer chains [44,57]. These additives make the PET-PCR unsuitable for contact with food. Solid-state polymerization (SSP) is also effective in reversing chain scission, thus contributing to the quality of the recycled material [44,57].

## 6. Legislation and Contaminants of PET-PCR

After the recycling process, recycled PET may contain a wide range of contaminants from various sources, which directly influence the quality and properties of the material [58], as well as the safety of recycled products for contact with food and beverages. Recycled PET has the potential to replace virgin PET and can be reused in similar products (closed loop) or in different categories (open loop) [59].

To meet the demand of the PET-PCR market for applications in food and beverages in Brazil, the National Health Surveillance Agency (ANVISA) published Technical Report No. 71/2016, which addresses the presence of contaminants in recycled plastic materials intended for food contact. These contaminants may include the presence of unauthorized compounds for use in food; incidental contaminants resulting from the improper reuse of packaging after consumption, such as the storage of pesticides and cleaning products; contamination of packaging due to the disposal environment; chemicals used during the recycling process; and substances originating from polymer degradation and additives applied in the manufacture of plastic packaging [60].

Thus, the presence of these contaminants not only compromises the efficiency of the recycling process but also raises concerns about toxicity and food safety in food packaging applications, as these components can migrate from the packaging to food and beverages. Therefore, it is essential to monitor and minimize contamination throughout the entire recycling chain to ensure a high-quality and safe final product [58].

### 6.1. Positive List and Migration Limits of Contaminants from Virgin PET and PCR

Brazilian legislation, through ANVISA Resolution No. 56/2012 [61], established the positive list, which comprises substances permitted and considered safe for use in PET packaging. This resolution sets parameters to ensure that there is no transfer of substances from the plastic material of the packaging to food at levels exceeding those allowed, thereby not posing a health risk, as these substances remain within the total and specific migration limits established for the packaging [62].

Additionally, Brazilian legislation includes a positive list of monomers for plastic materials in contact with food, as defined in RDC No. 56/2012 [61]. This regulation outlines the substances authorized for use in packaging intended for food contact and establishes the specific migration limits for these components. According to Technical Report No. 71/2016, PET-PCR packaging must provide documentation that ensures the compliance of the recycled resin with plastic materials legislation [60]. In this context, Table 1 describes the positive list of the main monomers used in the production of PET, as specified in RDC No. 56/2012 [61].

In addition to the substances described in Table 1, it is essential to evaluate other components in PET-PCR, including different monomers based on the polymer formulation. It is also important to analyze the specific migration of metals in colored packaging according to RDC n. 52/2010 [63], as well as the specific migration of primary aromatic amines in accordance with RDC n. 326/2019 [64]. Additionally, the migration of substances resulting from the use of additives in the manufacture of the precursor article or packaging should be considered, following the limits established by RDC’s n. 329/2019 and 589/2021 [64,65]. Finally, assessing the volatile profile is necessary to check for the possible presence of organic contaminants [66].

### 6.2. Requirements for the Safety of Food Contact Packaging

The Mercosur Technical Regulation on the Positive List of Monomers and Polymers establishes guidelines for the safety of plastic packaging intended for contact with food and beverages, including PET-PCR. This regulation imposes a rigorous evaluation of the safety of materials, focusing on the migration of undesirable contaminants, such as heavy metals and chemical substances that may be incorporated into the material during the recycling process [61].

Additionally, the positive list outlined in the regulation ensures that only monomers and polymers that have been carefully evaluated and considered safe are used in the manufacture of these packaging materials. Thus, this regulation is crucial to ensure that recycled products in contact with food and beverages meet stringent safety and quality standards [61].

According to RDC No. 51/2010, migration tests must be conducted using food simulants, which are solutions that mimic the properties of food. This procedure ensures an adequate evaluation of the safety and migration capacity of substances from the plastic packaging to the food. The use of simulants is essential to verify whether the material complies with the migration limits established by legislation, ensuring that there is no transfer of contaminants to food at levels that could pose health risks to consumers. Thus, food simulants allow for simulations under various conditions of use, such as time, temperature, and the chemical nature of the food, providing a more realistic and effective assessment of the safety of the final product [67].

According to Brazilian legislation, there are different types of simulants that can be used in testing. These are classified as food simulants A, B, C, D, and D’, corresponding to distilled or deionized water, a 3% (*m*/*v*) acetic acid solution in distilled or deionized water, a 10% ethanol solution in distilled or deionized water or the closest actual concentration of ethanol in the product (if it exceeds 10% (*v*/*v*)), a 95% (*v*/*v*) ethanol solution in distilled or deionized water (which can be replaced by isooctane or modified polyphenylene oxide), and edible oils or fats (such as olive oil, sunflower oil, corn oil) or synthetic triglyceride mixtures. The choice of simulant should be directly related to the type of food that will be packaged, as indicated in Table 2 and Table 3 [67].

To conduct migration tests, the plastic materials must be kept in contact with the food simulants for the predetermined time and temperature, in order to replicate the standard conditions of production, storage, distribution, marketing, and consumption of the food [67]. It is important to emphasize that these tests include the migration of intentionally added substances (IASs), such as monomers and additives. However, in addition to IASs, the packaging may also exhibit migration of non-intentionally added substances, known as NIASs. Furthermore, the recycling processes of PET-PCR need to be evaluated regarding the Challenge Test, which will be discussed in the next section.

### 6.3. Challenge Test of PET-PCR for Food Contact

The Challenge Test is a method used to assess the effectiveness of the decontamination process in the recycling of packaging. In this test, plastics intended for recycling are contaminated with reference substances, and the effectiveness of the decontamination is verified by measuring the residual concentration of these contaminants in the recycled material after all stages of the process [68]. This methodology is internationally recognized by the Food and Drug Administration (FDA) and the European Food Safety Authority (EFSA). To validate the test, it is checked whether the concentrations of the model contaminants are within the established limits. In Brazil, these contaminants, known as surrogates, have a limit of 220 µg/kg for food-grade PET-PCR, based on the maximum allowed in the human diet, which is 0.5 µg/kg of food [10].

The FDA recommends using contaminants with varying chemical and physical characteristics to simulate inappropriate plastic use by consumers. These contaminants should be readily accessible and include a polar non-volatile organic substance, a volatile non-polar organic substance, a polar non-volatile organic substance, a non-volatile non-polar organic substance, and a heavy metal salt. However, the latter is not recommended for PET, as the migration of a heavy metal surrogate to food has never been identified [12]. Table 4 presents the substances to be utilized in the Challenge Test, as outlined by American legislation and the corresponding regulations in Brazil.

The test procedure begins with the intentional contamination of a virgin bottle using model contaminants and a solvent like hexane for dilution. Alternatively, plastic flakes can be immersed in the contaminants at standard concentrations. The contaminated material should be stored and sealed for two weeks at 40 °C, with periodic stirring. After this period, the drained contaminants are collected, and the plastic is washed, measuring the content of each surrogate in the polymer. Finally, the polymer undergoes the recycling process, and the regenerated components or the material produced must be evaluated for residual contaminants, representing the worst-case scenario, assuming that every recycled product contains contaminants [12].

Furthermore, to ensure the quality of the produced PET-PCR, the applied technology, whether the Challenge Test or an equivalent, will only be valid if the process parameters remain constant and if the equipment used for decontamination is equivalent to the technology that was originally approved [10].

### 6.4. Requirements for Notification to ANVISA of Food-Grade PET-PCR Resin and Food-Grade PET-PCR Packaging

The notification regarding the use of PET-PCR resin and the packaging produced with this material, when intended for contact with food, must comply with the requirements established by Resolution RDC No. 843 and Instruction Normative IN No. 281, both dated 22 February 2024 [69,70]. The materials used in the manufacture of food-grade PET-PCR packaging may combine different proportions of virgin PET resin and food-grade PET-PCR. Therefore, it is essential to stay updated on the usage of this material to ensure compliance. Notification to ANVISA is mandatory in the following cases [66]:

(a) Recycling technology with high decontamination efficiency;
(b) Food-grade PET-PCR resin;
(c) Food-grade PET-PCR packaging or precursor article for food-grade PET-PCR packaging.

The prerequisites for notifying the recycling technology with high decontamination efficiency to ANVISA are as follows [66]:

(a) **Technology involved:** detailed description of the physical and/or chemical recycling technologies applied to the processing of post-consumer PET and/or industrial waste.
(b) **International background:** history of the use of these technologies in other countries, highlighting the regulations and practices adopted to ensure the quality of recycled PET.
(c) **Technology validation:** results of validation tests (such as challenge tests) demonstrating the effectiveness of the technology in removing contaminants, recognized by entities such as the FDA (USA) and EFSA (European Union).
(d) **Letters of no objection:** documents issued by agencies such as the FDA that attest to the safety of using food-grade PET-PCR resin, validating the technology and ensuring the safety of the recycled material for food contact.

Furthermore, producers of food-grade PET-PCR resin must implement a quality assurance system that includes the following: maintaining decontamination parameters, communicating any changes to the health authority, validating the effectiveness of the process, establishing analytical monitoring programs to ensure the quality of PET-PCR, conducting sensory analyses to verify the integrity of food characteristics, and maintaining records of raw materials and the final product. Notification for food-grade PET-PCR packaging or precursor articles requires a series of detailed documents and processes, including [66].

(a) **Process flowchart:** specification of the equipment and processes used in the manufacture of the packaging or precursor article.
(b) **Material specification:** details about the PET-PCR resin (supplier and notification to ANVISA), additives, and pigments used.
(c) **Type of packaging:** statement regarding the type of packaging to be produced and its conditions of use (single-layer, returnable, etc.).
(d) **Food specification:** details of the foods to be packaged and the percentages of PET-PCR resin, pigments, and additives.
(e) **Analysis reports:** results of total and specific migration (monomers, acetaldehyde, metals, aromatic amines) and volatile profile.
(f) **Notification form:** completed according to the ANVISA model.
(g) **Sanitary licensing:** document from the manufacturer proving compliance with the health authority.

Additionally, producers must comply with good manufacturing practices, record the origin of PET-PCR, control the process, and ensure traceability. The final packaging must be labeled as “PET-PCR” and indicate the manufacturer, in accordance with RDC No. 20/2008 [50].

Additionally, in the European Union, with the entry into force of Regulation (EU) 2022/1616, the EFSA has updated its guidelines to assist applicants in preparing requests related to the recycling of “post-consumer mechanical PET” for food contact applications. The guidelines establish evaluation criteria for the effectiveness of decontamination and require information about the recycling process, decontamination efficiency in tests with surrogate contaminants, and self-assessment. The residual concentration of contaminants in the recycled PET must remain below 3 mg/kg, ensuring that food exposure does not exceed 0.0025 μg/kg of body weight per day, a limit deemed safe. Based on this information, the EFSA will assess the safety of the recycling process [71].

### 6.5. Non-Intentionally Added Substances (NIASs) in PET-PCR

The migration of potentially hazardous substances from recycled packaging to food is a significant concern, not only for the food packaging sector but also for the personal care packaging industry, as it poses a potential risk to the end consumer. In this context, these unwanted chemicals are classified as non-intentionally added substances (NIASs) [72].

NIASs are unwanted chemical components that may be present in plastic material after recycling. They can have various origins, such as polymer degradation reactions and their additives, as well as contaminants from external sources. It is important to note that some of these non-targeted molecules can also be intentionally added substances (IASs) during the manufacturing process. For instance, when a package is printed, IASs can be introduced intentionally, which may become NIASs during the recycling process. Several studies are already focused on the identification and quantification of NIASs in post-consumer plastic packaging, such as bottles made from PET-PCR [72].

During recycling, materials undergo chemical treatments, heating, and degradation processes that can generate residual contaminants and byproducts, such as oligomers. These operations modify the mechanical and optical properties of plastics, resulting in the formation of oligomers, such as dimers and trimers, in recycled PET, as evidenced in studies mentioned in Table 5. Additionally, compounds such as plasticizers, antioxidants, stabilizers, and metals can lead to unwanted reaction products. The production and recycling of plastics can result in the accumulation of substances such as phthalates, toxic metals, brominated flame retardants, and polycyclic aromatic hydrocarbons [73]. These chemicals typically are not found in PET bottles, with the exception of plasticizers, which often originate from labels. The majority of these contaminants come exclusively from other plastic products, not from bottles themselves. Therefore, if the sorting process of the feedstock is efficient and effective, these chemicals will not enter the recycled PET, significantly reducing the potential for contamination and enhancing the overall quality and safety of the recycled material.

Other compounds responsible for flavor and aroma can also accumulate, including inks and ink residues, due to their low melting points. On the other hand, despite being more expensive, alkali-soluble adhesives are preferred because they facilitate disintegration in a 2% NaOH solution [9]. Surfactants and fatty substances play a significant role in accelerating the degradation of polymers. Additionally, residues of metallic catalysts present in recycled PET can trigger transesterification and polycondensation reactions, altering the chemical homogeneity of the material and compromising its rheological behavior during melting. The degradation of PET also results in the formation of acetaldehyde, a substance that can migrate into food, raising concerns about the safety of using PET-PCR in packaging [9].

Research shows that the benzene content detected in PET-PCR bottles increased proportionally with the percentage of PET-PCR in the bottles, originating from impurities in the recycled material. Additionally, acetaldehyde was produced in greater quantities during injection molding, where the material was exposed to higher temperatures, between 270 and 290 °C. In contrast, during the blow molding stage (110 °C), the production of acetaldehyde was lower. Medium- and low-volatility compounds were also found in the PET-PCR pellets and bottles. However, virgin PET bottles also exhibited high levels of oligomers, leading researchers to conclude that there is no direct relationship between the content of PET-PCR and its quality [74].

In the traditional recycling process, post-consumer PET is washed with water to remove dirt and labels, but organic substances, such as flavorings, may remain, making recycled PET unsuitable for contact with food, often redirecting it for the production of polyester fibers. In super-clean recycling processes, PET undergoes more intensive cleaning, with treatments at high temperatures, vacuum, or inert gas, along with non-toxic chemicals. These methods reduce contamination, allowing for the safe use of recycled PET in contact with food and beverages [75]. As previously mentioned, solid-state polymerization (SSP) is conducted on the pellets outside the extruder. One of the additional advantages of SSP is its ability to remove volatile compounds that were present prior to extrusion as well as those generated during the extruding process [54,76]. This technology is particularly beneficial for the production of food-grade PET-PCR in Brazil, ensuring a higher quality and safety of the final product.

**Table 5:** Main NIASs found in packaging made from PET-PCR.

Origin	Food/Food Simulant	NIAS	Highlights	References
Spain	3% (*m*/*v*) acetic acid solution in water. 10% (*v*/*v*) ethanol solution in water. 95% (*v*/*v*) ethanol solution in water.	Cyclic and linear oligomers.	- The results were comparable for both PET-PCR and virgin PET. - The highest migration potential for most of these oligomers was observed in a fatty food simulant (95% ethanol). - UPLC-MS-QTOF proved effective in determining the oligomer content in PET samples, as well as identifying non-volatile additives and other NIASs. - Six detected oligomers were categorized into the first series, five into the second series, and two into the third series.	[77]
Netherlands	Mineral water.	2-methyl-1,3-dioxolane, limonene, acetone, butanone, furan, benzene and styrene.	- The solvents acetone and butanone likely originate from agents used to clean and protect the mold. - Limonene is a natural compound widely used in orange-based beverages and detergents, and it can be detected even at extremely low concentrations in water. - No migration of benzene and styrene was observed in virgin PET bottles, while styrene was detected in PET-PCR, likely resulting from the thermal degradation of polystyrene contaminants.	[78]
Denmark	Parmesan cheese, sausages, roast chicken.	Acetophenone, benzophenone, 1-hydroxycyclohexyl-1-phenylketone, acetaldehyde, acetophenone, 2-methyl-1,3-dioxolane, benzene, styrene, hexadecenamide, edodecenamide and oligomers.	- Migration analysis revealed that trays made from different proportions of vPET/rPET can be distinguished by the presence of specific oligomers and other NIASs. - NIASs may originate from the degradation of the polymer derived from terephthalic acid or the removal of the ethylene glycol group. - The identified amides may arise from cleaning products, dyes, adhesives, sealants, and lubricants.	[79]
China	Recycled PET flakes with solvent.	Naphthalene-d8, dimethyl terephthalate, diisobutyl thalate, methyl stearate, bis(2-ethylhexyl) phthalate, benzothiazole, dimethyl phthalate, 1,2-diphenoxyethane, 2-hydroxyethylmethyl terephthalate, ethylene terephthalate cyclic dimer, benzene and substituted derivatives.	- PET-PCR samples did not employ the SSP process. - A non-target screening was conducted on 13 batches, totaling 39 samples of PET-PCR, using GC-QTOF-MS. - A total of 240 compounds were provisionally identified. - Variations in concentration among different batches may be attributed to factors such as the collection of post-consumer PET and the recycling processes involved.	[75]
Brazil	3% (*m*/*v*) acetic acid solution in water. 10% (*v*/*v*) ethanol solution in water. 95% (*v*/*v*) ethanol solution in water.	Bis(7-methyloctyl) hexanedioate, 1,2-benzenedicarboxylic acid diisononyl ester, 2,5-bis(5-tert-butyl-2-benzoxazolylthiophene, (Z)-octadec-9-enamide	- PET-PCR samples employed the SSP process. - The cleaning process applied to post-consumer PET samples significantly reduced the migration of non-volatile compounds. - However, none of the tested methods, including deep cleaning and super-cleaning, were able to completely eliminate the organic compounds present in the PET-PCR samples.	[80]

## 7. Future Perspectives for PET-PCR

The future perspectives for the use of PET-PCR are promising, driven by a growing trend toward sustainability and advanced recycling technologies. The expansion of chemical recycling processes will enable recycled materials to achieve performance comparable to that of virgin raw materials, representing a significant advance in the quality of recycled products. With the transition from laboratory applications to industrial scale, the adoption of advanced recycling technologies, such as the use of enzymes for PET hydrolysis, indicates potential for large-scale production [76,81,82].

Although Brazil still needs to make progress in regulating the sustainable disposal of post-consumer materials, tax incentives can encourage companies to incorporate PET-PCR into their packaging. This represents an opportunity for market growth and the adoption of more sustainable practices.

However, challenges persist, particularly the lack of adequate infrastructure and resources for recycling, especially in smaller areas where selective collection is scarce. To overcome these barriers, a joint effort among government, industry, and civil society will be crucial to establish an efficient recycling network and encourage active consumer participation.

Additionally, it is essential to stimulate the flow of selective collection, as the scarcity of PET-PCR in the market and its high cost hinder its adoption. By addressing these challenges, Brazil can not only enhance sustainability in the use of PET-PCR but also promote a circular economy and maximize the potential of this material across various sectors.

## 8. Conclusions

This review addresses the production and recycling cycle of PET, emphasizing recycling alternatives, with a focus on mechanical recycling, the most commonly used method. It also highlights the contaminants that may remain in PET after disposal and recycling, raising concerns for regulatory agencies, as these contaminants may be present in packaging made from recycled material. It is crucial to ensure that recycling and decontamination processes comply with current legislation, ensuring that the migration limits for contaminants are respected. This is vital to guarantee that recycled PET packaging intended for contact with food and beverages does not pose health risks to consumers. This validation is performed through the Challenge Test, which simulates contamination of the plastic material followed by recycling and checks for effective decontamination by assessing the migration limits of the contaminants. Finally, the studies mentioned emphasize the importance of evaluating the presence of NIASs in PET-PCR, aiming to enhance reverse logistics and the decontamination process in PET-PCR production. This ensures that the PET-PCR resins available on the market are safe for contact with food and beverages.

## Figures and Tables

**Table 1:** Specific migration limits (SMLs) of PET monomers [61].

Substance	Restriction or Specification
Terephthalic acid	SML = 7.5 mg/kg (expressed as terephthalic acid)
Isophthalic acid	SML = 5 mg/kg (expressed as isophthalic acid)
Dimethyl isophthalate	SML = 0.05 mg/kg
Mono- and diethylene glycol	SML = 30 mg/kg
Acetaldehyde	SML = 6 mg/kg

**Table 2:** Classification of foods according to Brazilian legislation [67].

Food Classification
Non-acidic aqueous foods (pH > 4.5)
Acidic aqueous foods (pH < 4.5)
Fatty foods (containing fat or oils among their components
Alcoholic foods (alcohol content greater than 5% (*v*/*v*)) Dry food

**Table 3:** Food simulant applied for each type of food [67].

Food Classification	Food Simulant
Only non-acidic aqueous foods	A
Only acidic aqueous foods	B
Only alcoholic foods	C
Only fatty foods	D or D’
Non-acidic and alcoholic aqueous foods	C
Acidic and alcoholic aqueous foods	B and C
Non-acidic aqueous foods containing fats and oils	A and D or D’
Acidic aqueous foods containing fats and oils	B and D or D’
Non-acidic, alcoholic and fatty aqueous foods	C and D or D’
Acidic, alcoholic and fatty aqueous foods	B, C and D or D’
Non-fatty dry foods	No migration test required
Fatty dry foods	D or D’

**Table 4:** Contaminants indicated for the Challenge Test [12].

Characteristic	Substance
Volatile and polar	Chloroform Chlorobenzene 1,1,1-Trichloroethane Diethyl ketone
Volatile and non-polar	Toluene
Heavy metal	Copper (II) 2-ethyl hexanoate
Non-volatile and polar	Benzophenone Methyl salicylate
Non-volatile and non-polar	Tetracosane Lindane Methyl stearate Phenyl cyclohexane 1-Phenyldecane 2,4,6-Trichloroanisole

## Data Availability

Not applicable.
